# Attack rate and the price of SARS-CoV-2 herd immunity in Brazil

**DOI:** 10.21203/rs.3.rs-659187/v2

**Published:** 2021-07-08

**Authors:** Tarcisio Rocha Filho, José Mendes, Carson Chow, James Phillips, Antônio Cordeiro, Fulvio Scorza, Antonio-Carlos Almeida, Marcelo Moret

**Affiliations:** Universidade de Brasilia; UNiversity of Aveiro; NIH/NIDDK; Rutgers Univ; Centro Universitário SENAI CIMATEC and Instituto Federal de Educação, Ciência e Tecnologia da Bahia and Unopar Candeias; Universidade Federal de Sao Paulo; Universidade Federal de São João del Rey; Centro Universitário SENAI CIMATEC

**Keywords:** SARS-COV-2, herd immunity, Brazil

## Abstract

We introduce a compartmental model with age structure to study the dynamics of the SARS-COV-2 pandemic. The contagion matrix in the model is given by the product of a probability per contact with a contact matrix explicitly taking into account the contact structure among different age groups. The probability of contagion per contact is considered as time dependent to represent non-pharmaceutical interventions, and is fitted from the time series of deaths. The approach is used to study the evolution of the COVID-19 pandemic in the main Brazilian cities and compared to two good quality serological surveys. We also discuss with some detail the case of the city of Manaus which raised special attention due to a previous report of three-quarters attack rate by the end of 2020. We discuss estimates for Manaus and all Brazilian cities with a total population of more than one million. We also estimate the attack rate with respect to the total population, in each Brazilian state by January, 1^st^ 2021 and May, 23 2021.

## Introduction

**Figure 1:**
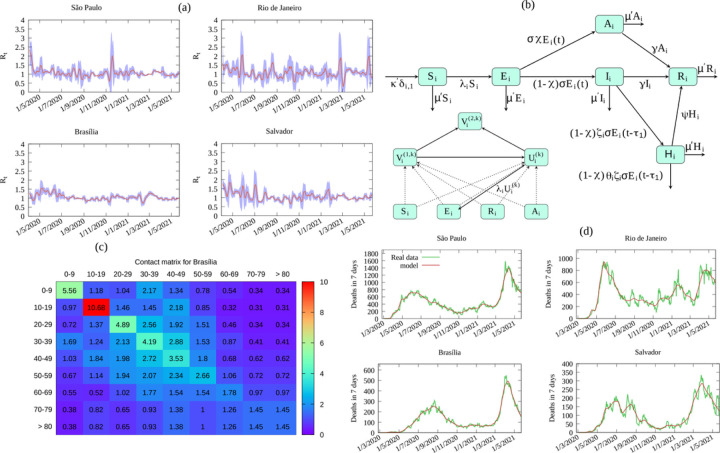
a) Reproduction number Rt and 95% confidence interval (shaded region) for the four most populous Brazilian municipalities. b) Diagrammatic representation of the vaccination processes in the epidemiological model. Dotted lines indicate a flow between different compartments due to vaccination, and according to the effectiveness of each vaccine type with one and two doses. d) Estimated contact matrix for the capital Brasília. d) Epidemic curves from real and model data.

The present COVID-19 pandemic initiated in the end of 2019 in China, and was first reported to the World Health Organization in January 2020 as an outbreak of pneumonia, of undetermined origin, in the city of Wuhan and with etiologic agent latter identified as the the new coronavirus SARS-CoV-2 [1]. The resulting disease caused until the present day more than 3.9 million deaths in the whole world, with a reported number of 180 million cases [2]. Different waves of the pandemic occurred at various moments of time and in almost all countries of the world. Mitigation measures were widely adopted such as social distancing and mask wearing. A few vaccines were recently developed and approved, with few countries having vaccinated a significant part of the population [3]. Since the beginning of 2021 Brazil has received great attention due to a strong second wave, resulting in overcrowded hospitals, a lack of basic medical supplies for a proper treatment in ICUs, and people dying asphyxiated with lack of oxygen [4]. The time evolution of the reproduction number R_t_ from the official number of cases for the four more populous cities in Brazil is shown in Fig. 1a, remaining above 1 for a significant portion of the time. The lack of a centralized coordinated effort to mitigate the pandemic allowed for a rapid circulation of the virus, in a country with the sixth largest population in the world, resulting in one more hotbed for variants. After more than a year of pandemic some variants of concern have emerged: a (B.1.1.7 from the United Kingdom), b (B.1.351 from South Africa), g (P1 from Brazil), e (B.1.427–9 from the US), and more recently the d variant, from India, which in now prevalent in the UK. Those variants are 1.5 to 2.5 more transmissible than the original strain, and possibly able to reinfect individuals with a former infection by the original variant [4,31].

The official figures for the total number of cases, and also deaths although to a lesser extent, are underestimated all over the world [5,6], and particularly in Brazil [7] due to a very small number of tests per million inhabitants (246 782), at the 118-th position among all countries [8]. A realistic estimate of the number of people already infected by the virus is of uttermost importance for implementing and evaluating mitigation policies, such as social distancing with different intensities, as well as for prediction of possible outcomes of the pandemic in each locality. For that purpose serological surveys for detecting SARS-CoV-2 antibodies were implemented in many countries worldwide, with most of them of low quality [9]. Among the minority of high quality surveys there are two in Brazil: an estimate of the attack rate in the state of Rio Grande do Sul, based on a serological survey in ten municipalities [10], and the SoroEpi MSP in the city (municipality) of São Paulo [11]. The former covered a time span only from from April, 1 to May, 11 2020, while the survey in São Paulo reported five surveys from April 2020 to January 2021.

When good quality serological surveys are not available, alternative methods must be used to estimate realistic attack rates. We present here a model based approach using available data on the total number of deaths and epidemiological parameters. We first tested our approach with the surveys for the municipality of São Paulo, the most populous city in the southern hemisphere, and the Brazilian state of Rio Grande do Sul. We then apply it to the main Brazilian cities, with a total population over one million inhabitants, and discuss the special case of the highly impacted city of Manaus, in the Amazon region. We also present rough estimates for the attack rate for each Brazilian state and discuss the implications of pursuing natural herd immunity in the current context.

## Determination Of Sars-cov-2 Attack Rate

We use an age structured model model with different compartments for Susceptible (S), Exposed (E), Infected (I), Asymptomatic (A), Hospitalized (H), Recovered (R), Vaccinated individuals with one (V(1)) and two (V(2)) doses without primary vaccination failure, and a compartment (U) for vaccinated individuals with primary vaccination failure, considering different vaccine types and efficacies. A diagrammatic representation of the model is given in Fig. 1b and the corresponding equation in Eq. (S1) (see methods and supplementary information for model details on the model and the fitting from data, and parameters used in the model). The model is fitted to reproduce the time series of deaths by COVID-19 as shown in Fig. 1d for the four more populous cities in Brazil. COVID-19 and demographic data was obtained from Brazilian government sources [12–14], and epidemiological parameters [14–21] (see methods and supplementary information for model details and parameter values). All data considered here span the period from February, 26 2020 (first case in Brazil) up to May, 23 2021.

**Figure 2:**
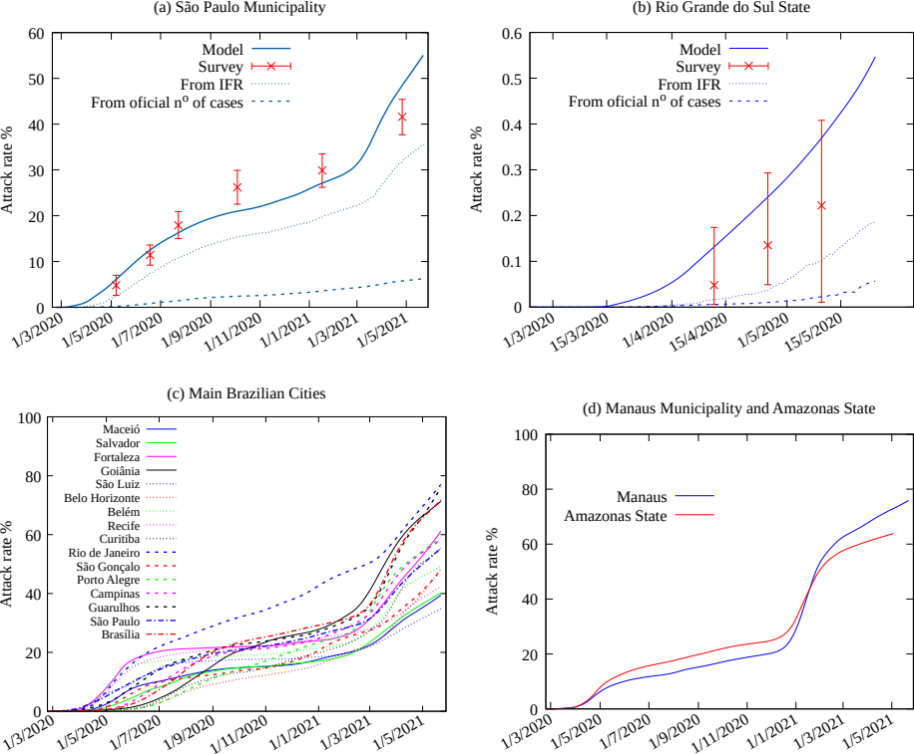
a) Attack rate from the total number of cases (symptomatic and non-symptomatic) as obtained from the epidemiological model (solid line), and data from the SoroEpi MSP serological survey [11]. The dotted line gives the total number of cases estimated from the average IFR using Eq. (S4), and the dashed line is obtained from the official number of cases as reported by the Brazilian Ministry of Health [12]. b) Attack rate for the state of Rio Grande do Sul from the epidemiological model and IFR estimates, official number of cases and serological survey [10]. c) Model estimates for the attack rate for Brazilian cities with more than one million inhabitants. d) Model estimates for the attack rate for the Amazonas state and Manaus municipality.

The attack rate is determined from the model output for the total number of cases. We first compare the results for our model with the surveys for São Paulo and Rio Grande do Sul. The results are shown in Fig. 2a and 2b, alongside official values, the results from the serological surveys and estimates obtained from the averaged Infection Fatality Ratio (IFR) as given by Eq. (S4). The serological survey for the Rio Grande do Sul state was obtained from data for the ten largest cities in the state, and is not necessarily representative of the whole state [10], while the time span considered corresponds to the very beginning of the pandemic in the state and a very low number of cases and deaths, which resulted in greater estimated errors. Results from our model for the city of São Paulo are in a good agreement with the SoroEpi MSP survey. Pearson correlation coefficient R, the p-value and mean square error for the model estimates, and estimates obtained from the average IFR, are given in table (S1). We note that SEIAHRV model considers explicitly the social contact structure expressed by the contact matrix among individuals of different age groups and thus is a better representative of how the virus spreads than a simple IFR average.

The model predicts a higher attack rate for the city of São Paulo at the end of April 2021 compared to the serological survey. A possible explanation being that emerging variants may result in an increase in mortality, and thence an overestimation of the total number of cases [22–24]. A straightforward calculation using the serological survey confidence interval indicates that the death rate has increased by 6% to 16% in the municipality of São Paulo during the first quarter of 2021. Another possible explanation for this is the overwhelming of the local health system. Nevertheless, this is expected to be less important in São Paulo, with the best health infrastructure in the country, than in other regions of Brazil.

## Main Brazilian Cities

The time evolution of the attack rate obtained from the model for Brazilian cities with a population of more than one million (see Table S6) is shown in Fig. 2c. The fatality ratio in ICUs varies according to the region in Brazil, from 49% to 79% in the southeast and north regions, respectively [25]. The IFR values for each age group are then corrected accordingly, with the IFR in the Southeast region, the one with the better health infrastructure, as given in table S3. The resulting attack rates are shown in Fig. 2c. Results for Manaus and the Amazonas state are given in Fig. 2d, and discussed with greater attention below.

### The case of Manaus

The attack rate for the city of Manaus and for the Amazonas state in May, 23 2021 are 77.1% and 54.9%, respectively. This result must be interpreted with due care. The Amazonas state is the largest state in Brazil, with 1 559 146 Km^2^, 18% of the Brazilian territory, but only 2% of its population. The state capital Manaus has 2 219 580 inhabitants, a little more than half the population of the state. The second largest city has a population of only 101 thousand, and almost all medical facilities, including ICUs, are in Manaus. As a consequence most of the critical patients, with a higher probability of dying, are transferred there. This explains why 69% of the deaths in the state occurred in Manaus, while only 46% of the official number of cases were reported there. This flow of critical cases to Manaus was even more pronounced during the second wave, the deadliest observed in Brazil until now, which overwhelmed all medical facilities. Therefore, fitting the model with the time series of deaths in Manaus is unrealistic. The attack rate in the city is most probably closer to the one estimated for the whole Amazonas state. Such peculiarities do not occur, at least not in the same intensity, for the other cities considered here. The occurrence of the P1 variant in the Amazonas state could also explain a higher value of the attack rate due to possible reinfections, as claimed in [26], but not the asymmetry of the number of deaths compared to the total number of cases between Manaus and the rest of the state.

A previous estimate by Bussi et al [27], based on a survey of samples from blood donors, obtained an attack rate of 76% in October, 1^st^ 2020 in Manaus. Therefore the important second wave that occurred in January 2021 would imply a high proportion of reinfection among new cases, possibly caused by the more contagious P1 variant. Nevertheless this value for the attack rate is highly at variance with our finding of 28.6% for the whole state and 39.6% for the city of Manaus in January, 15 2021. This significant difference can be explained out by two facts: Bussi and collaborators considered a model for seroconversion with an exponential decay to estimate the expected real number of individuals already infected. Although the results obtained for São Paulo are close to those from the SoroEpi MSP survey, we note that small errors are exponentially increased, which could lead to large errors in the estimate, while the SoroEpi MSP survey relied on a blood test with negligible seroconversion during the time span considered. The second fact is that using samples from blood donors introduces a strong bias, which is even more important if one considers that blood donations in the state of Amazonas are only possible in Manaus, and that the family of hospitalized individuals are often asked to donate blood, and that it is a common practice for people from other Amazonas cities to register in the hospital using the address of a relative living in Manaus. As a consequence, many donors in Manaus have been in recent contact with hospitalized individuals from all over the state.

### Brazilian states

**Figure 3:**
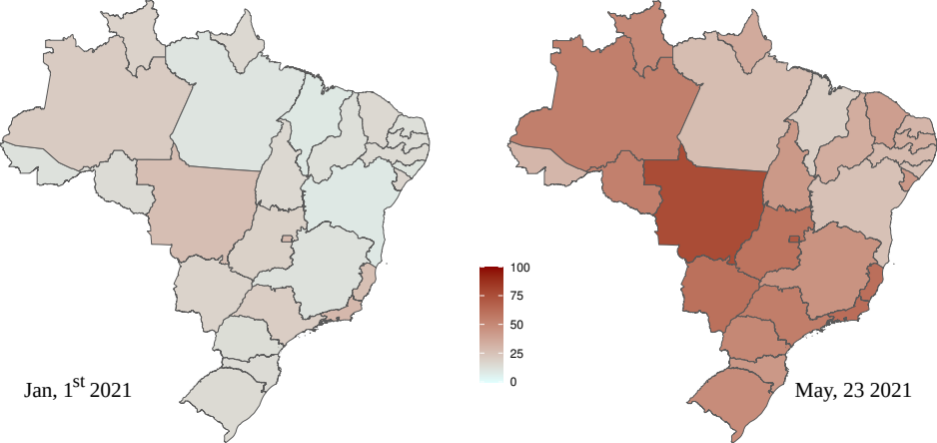
Heat map with the model estimates of the attack rate in each Brazilian state. The estimates for May, 23 2021 must be considered with care due to a possible increase in mortality during the second wave in Brazil (see text).

Estimates for the attack rate in each Brazilian state by considering the time series for the cumulative number of deaths for the whole state. Population and COVID-19 data are obtained from the same sources. Results for January, 1^st^ and May, 23 2021 are shown in Tables S8 and S9, respectively, and represented as heat maps in Fig. 3. We perceive that the second wave during the first months of 2021 was indeed very important, but with different intensities in each state. This an important information for guiding future mitigation policies.

## Discussion And Conclusions

We have shown that the attack rate estimated for most of Brazil is still well below herd immunity, estimated at the initial stages of the pandemic close to 70% [28], but at the present stage expected to have a higher value due to new and more transmissible variants. On the other hand, some states and cities are approaching this value. Supposing that the proportion of new cases due to reinfections is not high up to the present point, which is reinforced by the fact that no reports of a significant proportion of reinfections exist, it may explain why the pandemics is decreasing, or at least not increasing significantly, in some of the most struck states, as Amazonas, Distrito Federal and Mato Grosso, notwithstanding poor mitigation measures, while the situations is still critical in those with a lower attack rate. Nevertheless, it is difficult to correlate the effective reproduction number and the attack rate at this geographical level. It is expected that future results of serological surveys can shed some light on the possibility of a greater proportion of reinfections, and we hope that the present approach can be used as an important tool in this analysis. Our approach can be extended straightforwardly to other cities and countries with reliable data. This is usually the case for the number of deaths but not for the number of cases as under-reporting is important almost anywhere [5–7].

It is yet not clear how long natural immunity will last, and how much current vaccines protect against transmission. This situation become more complex with the emergence of new variants [22,23,26], particularly with the possible arrival of the even more contagious d variant [31], and thence casting many doubts on the real relevance of herd immunity. The determination of the attack rate is important for guiding, and for correcting public policies as a criterion to estimate its results, particularly by showing the high cost resulting from any expectation to attain herd immunity by other means than vaccination.

## Methods

### Data sources

All COVID-19 data is publicly available at the Brazilian Health Ministry COVID-19 website [12]. Estimates of population by age-group for 2020 are available at the Brazilian Institute for Geography and Statistics [20] and from DATASUS system of the Ministry of Health [12]. Data on the EPI-MSP serological survey in the São Paulo municipality, with additional details on the survey, is available in the SoroEpi MSP Website [11]. Data on the Rio Grande do Sul state serological survey is reported in the paper in [10]. Detailed data on each vaccine shot applied in Brasil is publicly available on the at Brazilian Health Ministry [13]. All data are publicly available.

### Epidemiological model

We use an extension of the model described in [32], the SEIAHRV model (Susceptible, Exposed, Infected, Asymptomatic, Hospitalized Recovered and Vaccinated), and additional compartments for individuals vaccinated with one and two doses, V^(1,k)^ and V^(2,k)^ for vaccine of type k, respectively, without primary vaccination failure, and for vaccinated individuals with primary vaccination failure U. The model assumes homogeneous mixing and M age groups. The variables are given as proportion with respect to the total population at the initial time. The decomposition of the compartments into age groups enables to incorporate the estimated contact structure in a given population, represented here by a contact matrix C_i,j_, as the average number of contacts per day of a single individual of age group j with any individual of age group i (see supplementary material for the estimation of Ci,j). All variables in the model are proportions with respect to the initial population N_0_ (the present population changes due to mortality and birth). The model considers nine age groups: 0 to 9, 10 to 19, 20 to 29, 30 to 39, 40 to 49, 50 to 59, 60 to 69, 70 to 79 and 80 years of age and more. Given n_i_ the proportion of the population in age-group i and ξ the infectiousness of an asymptomatic individual with respect to a symptomatic one, the force of infection is given by see formula 1 in the supplementary files where b_i,j_=p_c_ C_ij_ is the infection rate matrix, with p_c_ the probability of contagion per single contact, assumed to be age independent. Required parameters are given in Tables S2 and S3. The model dynamical system of ordinary differential equations with delays is given in Eq. (S1) and its diagrammatic representation in Fig. 1b. The probability of contagion pc is fitted using the time series of total deaths reported in each locality a piece-wise function of time with 14 days intervals (see supplementary material for details). This time varying contagion probability is used to represent changes in behavior, social distancing, and other mitigation policies. Figure 1d shows the resulting fit for the four main Brazilian cities.

## Supplementary Material

Supplement 1
