# Supplementary material for: Attack rate and the price of SARS-CoV-2 herd immunity in Brazil
Source: Res Sq. 2021 Jul 8:rs.3.rs-659187. Preprint. [Version 2] doi: 10.21203/rs.3.rs-659187/v2 (PMC8282102; doi:10.21203/rs.3.rs-659187/v2)
Supplement: Supplement 1 [file 2c3f87938db6d489370085e8.docx]

**Attack rate and the price of SARS-CoV-2 herd immunity in Brazil - Supplementary material**

# The SEIAHRV model

We use an extension of the model described in [17], the SEIAHRV model (Susceptible, Exposed, Infected, Asymptomatic, Hospitalized Recovered and Vaccinated), and compartments for individuals vaccinated with one and two doses, V^(1,k)^ and V^(2,k)^, respectively for vaccine of type k, without primary vaccination failure, and vaccinated individuals with primary vaccination failure U, with homogeneous mixing and M age groups. The variables are given as proportion with respect to the total population at the initial time. The decomposition of the compartments into age groups allows to incorporate the contact structure in a given population, represented in the our model by a contact matrix. All variables in the model are proportions with respect to the initial population N_0_ (the present population changes due to mortality and birth). The model considers nine age groups: 0 to 9, 10 to 19, 20 to 29, 30 to 39, 40 to 49, 50 to 59, 60 to 69, 70 to 79 and 80 years of age and more. Given n_i_ the proportion of the population in the age-group i, and ξ the infectiousness of asymptomatic with respect to symptomatic individuals, the force of infection is given by $\lambda_{i}=\sum_{j=1}^{M} \beta_{i,j}\left( I_{j}+\xi A_{j} \right)/{n_{i}},$ and depends on the infection rate matrix $\beta_{i,j}=p_{c}C_{i,j}$, with p_c_ a probability of contagion per single contact, assumed to be age independent, and C_i,j_ the contact matrix giving the average number of contacts per day of a single individual of age group j with any individual of age group i. Required epidemiological parameters are given in Tables S1 and S2. The diagrammatic representation of the model is given in Fig. 1B, and corresponding dynamical system of diffrenetial equations with time delay is:

$$\frac{dS_{i}}{dt}=-\left[ \lambda_{i}+\mu'+\nu_{i}+\frac{1}{p_{v,i}}\sum_{k=1}^{K} d_{1}^{\left( i,k \right)} \right]S_{i}+\kappa'\delta_{i,1}+\nu_{i-1}S_{i-1},$$

$\frac{dE_{i}}{dt}=\lambda_{i}S_{i}+\lambda_{i}\sum_{k=1}^{K} U_{i}^{\left( k \right)}-\left[ \sigma+\mu'+\nu_{i}+\frac{1}{p_{v,i}}\sum_{k=1}^{K} d_{1}^{\left( i,k \right)}e_{1}^{\left( k \right)} \right]E_{i}+\nu_{i-1}E_{i-1},$ $\frac{dI_{i}}{dt}=\left( 1-\chi\right)\sigma E_{i}-\left[ \gamma+\mu'+\nu_{i} \right]I_{i}+\nu_{i-1}I_{i-1}-\left( 1-\chi\right)\zeta_{i}\sigma E_{i}\left( t-\tau_{1} \right),$ $\frac{dA_{i}}{dt}=\chi\sigma E_{i}-\left[ \gamma+\mu'+\nu_{i}+\frac{1}{p_{v,i}}\sum_{k=1}^{K} d_{1}^{\left( i,k \right)}e_{1}^{\left( k \right)}A_{i} \right]+\nu_{i-1}A_{i-1},$

$$\frac{dH_{i}}{dt}=-\left[ \psi+\mu'+\nu_{i} \right]H_{i}+\left( 1-\chi\right)\zeta_{i}\sigma E_{i}\left( t-\tau_{1} \right)-\left( 1-\chi\right)\theta_{i}\zeta_{i}\sigma E_{i}\left( t-\tau_{2} \right)+\nu_{i-1}H_{i-1},$$

$\frac{dR_{i}}{dt}=\gamma I_{i}+\gamma A_{i}+\psi H_{i}-\left[ \mu'+\nu_{i}+\frac{1}{p_{v,i}}\sum_{k=1}^{K} d_{1}^{\left( i,k \right)}e_{1}^{\left( k \right)} \right]R_{i}+\nu_{i-1}R_{i-1},$ $\frac{dV_{i}^{\left( 1,k \right)}}{dt}=-\left[ \mu'+\nu_{i} \right]V_{i}^{\left( 1,k \right)}+\nu_{i-1}V_{i-1}^{\left( 1,k \right)}+e_{1}^{\left( k \right)}d_{1}^{\left( i,k \right)}-e_{1}^{\left( k \right)}d_{2}^{\left( i,k \right)},$

$$\frac{dV_{i}^{\left( 2,k \right)}}{dt}=-\left[ \mu'+\nu_{i} \right]V_{i}^{\left( 2,k \right)}+\nu_{i-1}V_{i-1}^{\left( 2,k \right)}+e_{2}^{\left( k \right)}d_{2}^{\left( i,k \right)},$$

$\frac{dU_{i}^{\left( k \right)}}{dt}=-\left[ \lambda_{i}+\mu'+\nu_{i} \right]U_{i}^{\left( k \right)}+\nu_{i-1}U_{i-1}^{\left( k \right)},$ (S1)

with p_v,i_=S_i_+E_i_+A_i_+R_i_, d_1_^(i,k)^ and d_2_^(i,k)^ the number of doses for the first and second shot, respectively, per unit of time for the age group i, of vaccine type k, e_1_^(k)^ and e_2_^(k)^ the efficacy of vaccine type k with one and two shots, respectively, k=1,…,K, with K the number of different vaccines, μ’=μN/N_0_ and κ’=κN/N_0_, with N_0_, N, κ and μ the initial population, current population, birth and natural mortality rates, respectively, with the mortality rate distributed homogeneously through all age groups. The aging rate from i-th to the (i+1)-th age group is denoted by $\nu_{i}$ and given by the inverse of the time span of the i-th age group, with ν_0_=0. The force of infection λ_i_ on the i-th age group is given by

$\lambda_{i}=\sum_{j=1}^{M} \beta_{i,j}\left( I_{j}+\xi A_{j} \right)/{n_{i}},$ (S2)

where n_i_ is the proportion of the population in the i-th age group with respect to the current population, and the transmission probability to a susceptible individual of age group i from an infected individual of age group j is given by β_i,j_=p_c_C_i,j_, with p_c_ the probability of contagion on a single contact, supposed to be time dependent and the same for all age groups, and C_i,j_ the contact matrix that gives the average number of contacts per day of a single individual of group j with any individual of group i.

Up to the authors knowledge there is no estimation for the contact matrix in Brazil, so we use the estimate by Mossong et al. [32], averaged over eight European countries and adapted to the age distribution in each locality considered. This is a reasonable assumption due to cultural similarities between Europe and Brazil. The probability of contagion is determined from real data, as we explain below. The model is solved using a fourth-order Runge-Kutta method and is implemented in the C language. The code is available from T.M.R.F. on demand.

For the current COVID-19 pandemic the number of cases is not a reliable as under-reporting is common and high [6]. Although also subject to some under-reporting the number of deaths is much closer to reality [7,8]. In the present work we fit the time varying parameter p_c_ by adjusting the model output for the cumulative number of deaths to the publicly available data. We use a step-function such that p_c_ is constant in intervals of 14 days, and varying from interval to interval. We consider that the virus spread starts at time zero from only a number of individual in the last age group, which is also a parameter to be fitted. This level values for p_c_ are determined by minimizing the error function:

$E=\sqrt{\frac{1}{N_{data}}\sum_{i=1}^{N} \left( D_{i}^{\left( r \right)}-D_{i}^{\left( m \right)} \right)^{2}},$ (S3)

where N is the number of data points to fit, $D_{i}^{\left( r \right)}$ and $D_{i}^{\left( m \right)}$ are the real and model values, respectively. The minimization procedure is implemented using a simplex algorithm with adaptative parameters [33]. The number of cases and deaths by COVID-19 in each state and municipality in Brazil are available online at the Brazilian Health Ministry [15]. Each compartment of the model is divided in M=9 age groups specified in Table S2, which parallels known data on the Infection Fatality Rate (IFR) [18]. Population data for each age group considered are from official estimates for 2020 [16].

**Estimating the total number of cases from the average Infection Fatality Ratio**

A more direct estimate of the number of cases can be obtained from the statistical distribution F_d_ for the time between the first symptom and death is given by [21]:

$c\left( i \right)=\frac{1}{L_{avg}}\sum_{j=14}^{22} d\left( i+j \right)F_{d}\left( j \right)$ (S4)

with c(i) and d(i) the numbers of new cases and deaths in day i, respectively, and L_avg_ the average IFR for the given distribution of population among age groups, which for São Paulo is L_avg_=0.0053 and L_avg_=0.0084 for Rio Grande do Sul. Population estimates for each locality and each age group are given in Tables S3 and S4.

**Epidemiological parameters**

The different epidemiological parameters used in the model are given in tables S1 and S2 below, the index i referring to the age group;

| **Parameter** | **Definition** | **Value (IC** $95\text{\%}$**) [Ref]** |
| --- | --- | --- |
| $\psi$ | Recovery rate from hospitalization | 1/17.5 days^-1^ [18,19] |
| $\sigma$ | Inverse of incubation time | 1/5.0 days^-1^ [20] |
| $\gamma$ | Recovery rate for non hospitalized  individuals | 1/3.69 days^-1^ [21] |
| $\theta_{i}$ | Fatality rate among hospitalized individuals | ${L_{i}^{\left( 0 \right)}}/{\zeta_{i}}$ (see table S2) |
| $\tau_{1}$ | Median time from first symptoms to hospitalization | 3.3 [20] |
| $\tau_{2}$ | Average time from first symptoms to death | 16.8 days [18] |
| $\chi$ | Proportion of asymptomatic cases | 17.9% [22] |
| $\xi$ | Contagiousness of asymptomatic with respect to symptomatic individuals | 55% [21] |
| $\kappa$ | Average birth rate in Brazil | 1.416% [15] |
| $\mu$ | Average natural mortality rate in Brazil | 0.608% [15] |

**Table S1:** Epidemiological parameters in the model.

| Age Group | IFR | $\zeta_{i}$ |
| --- | --- | --- |
| 0 – 9 | 0.0% | 0.00161% |
| 10 – 19 | 0.2% | 0.408% |
| 20 – 29 | 0.2% | 1.04% |
| 30 – 39 | 0.2% | 3.43% |
| 40 – 49 | 0.4% | 4.35% |
| 50 – 59 | 1.3% | 8.16% |
| 60 – 69 | 3.6% | 11.8% |
| 70 – 79 | 8.0% | 16.6% |
| 80 | 14.8% | 18.4% |

**Table S2:** Infection fatality ratio (IFR) $L_{i}^{\left( 0 \right)}$ and hospitalization probability $\zeta_{i}$ for each age group as obtained by Linton et al. [19].

# Population data

Population figures presented below are official estimates for 2020 and are available at [14,15].

| **Age group** | 0 – 9 | 10 – 19 | 20 – 29 | 30 – 39 | 40 – 49 |
| --- | --- | --- | --- | --- | --- |
| **Population** | 1 569 846 | 1 641 743 | 1 827 397 | 2 044 130 | 1 815 252 |
| **Age group** | 50 – 59 | 60 – 69 | 70 – 79 | 80 |  |
| **Population** | 1 443 720 | 1 083 124 | 589 507 | 310 513 |  |

# Table S**3**: Age groups and respective population for the city of São Paulo.

| **Age group** | 0 – 9 | 10 – 19 | 20 – 29 | 30 – 39 | 40 – 49 |
| --- | --- | --- | --- | --- | --- |
| **Population** | 1 403 318 | 1 437 763 | 1 721 149 | 1 726 904 | 1 519 290 |
| **Age group** | 50 – 59 | 60 – 69 | 70 – 79 | 80 |  |
| **Population** | 1 470 842 | 1 166 751 | 650 898 | 326 058 |  |

**Table S4:** Age groups and respective population for the state of Rio Grande do Sul.

| **City** | **Population** | **City** | **Population** |
| --- | --- | --- | --- |
| Maceió | 1 018 948 | Curitiba | 1 933 105 |
| Manaus | 2 182 763 | Rio de Janeiro | 6 718 903 |
| Salvador | 2 872 347 | São Gonçalo | 1 084 839 |
| Fortaleza | 2 669 342 | Porto Alegre | 1 483 771 |
| Goiânia | 1 516 113 | Campinas | 1 204 073 |
| São Luís | 1 101 884 | Guarulhos | 1 379 182 |
| Belo Horizonte | 2 512 070 | São Paulo | 12 252 023 |
| Belém | 1 492 745 | Brasília | 3 055 149 |
| Recife | 1 645 727 |  |  |

**Table S5:** Brazilian municipalities with a population over one million and the respective total population.

| **Age group** | 0 – 9 | 10 – 19 | 20 – 29 | 30 – 39 | 40 – 49 |
| --- | --- | --- | --- | --- | --- |
| **Population** | 1 403 318 | 1 437 763 | 1 721 149 | 1 726 904 | 1 519 290 |
| **Age group** | 50 – 59 | 60 – 69 | 70 – 79 | 80 |  |
| **Population** | 1 470 842 | 1 166 751 | 650 898 | 326 058 |  |

**Table S6:** Age groups and respective population for the state of Rio Grande do Sul.

**Attack rate in each Brazilian state**

| **State** | **AR %** | **State** | **AR %** | **State** | **AR %** |
| --- | --- | --- | --- | --- | --- |
| Acre | 12.2 | Maranhão | 9.8 | Rio de Janeiro | 30.1 |
| Alagoas | 11.9 | Mato Grosso | 27.5 | Rio Grande do Norte | 13.3 |
| Amapá | 16.2 | Mato Grosso do Sul | 18.6 | Rio Grande do Sul | 15.5 |
| Amazonas | 21.9 | Minas Gerais | 12.5 | Rondônia | 14.9 |
| Bahia | 9.6 | Pará | 11.1 | Roraima | 18.9 |
| Ceará | 16.5 | Paraíba | 14.3 | Santa Catarina | 14.9 |
| Distrito Federal | 29.1 | Paraná | 14.4 | São Paulo | 21.0 |
| Espírito Santo | 26.8 | Pernambuco | 15.5 | Sergipe | 17.5 |
| Goiás | 19.5 | Piauí | 13.5 | Tocantins | 16.4 |

**Table S7:** Attack rate estimated in each Brazilian state at January, 1^st^ 2021.

| **State** | **AR %** | **State** | **AR %** | **State** | **AR %** |
| --- | --- | --- | --- | --- | --- |
| Acre | 30.8 | Maranhão | 20.7 | Rio de Janeiro | 62.6 |
| Alagoas | 26.5 | Mato Grosso | 77.1 | Rio Grande do Norte | 31.2 |
| Amapá | 36.1 | Mato Grosso do Sul | 61.2 | Rio Grande do Sul | 49.1 |
| Amazonas | 54.9 | Minas Gerais | 46.2 | Rondônia | 54.3 |
| Bahia | 26.1 | Pará | 28.0 | Roraima | 50.9 |
| Ceará | 41.1 | Paraíba | 34.4 | Santa Catarina | 43.9 |
| Distrito Federal | 71.6 | Paraná | 51.0 | São Paulo | 55.5 |
| Espírito Santo | 61.8 | Pernambuco | 29.2 | Sergipe | 44.0 |
| Goiás | 59.9 | Piauí | 34.8 | Tocantins | 43.6 |

**Table S8:** Attack Rate (AR) estimated in each Brazilian state on May, 23 2021. These values are possibly overestimated due to an expected increase in mortality due to the overwhelming of the health system during a pronounced second wave in 2021. An increase in mortality due to new variants is also a possibility.

**Supplementary references:**

[32] Mossong J, Hens M, Jit M, Beutels P, Auranen K, Mikolajczyk R, Massari M et al. *Social Contacts and Mixing Patterns Relevant to the Spread of Infectious Diseases.* PLOS Medicine 2008;**5**:e74. Available at: h[ttps://doi.org/10.1371/journal.pmed.0050074](https://doi.org/10.1371/journal.pmed.0050074)

[33] Gao F, Han L. *Implementing the Nelder-Mead simplex algorithm with adaptive parameters*. Comput. Optim. Appl. 2012;**51**:259-277. Available at https://doi.org/10.1007/s10589-010-9329-3
